# EMPLOYING DESIGN THINKING METHODS TO CREATE BEHAVIORAL INTERVENTIONS THAT SUPPORT AFRICAN AMERICAN CAREGIVERS

**DOI:** 10.1093/geroni/igac059.1152

**Published:** 2022-12-20

**Authors:** Leila Aflatoony, Kenneth Hepburn, Molly Perkins, Drenna Waldrop, Karah Alexander

**Affiliations:** Georgia Institute of Technology, Atlanta, Georgia, United States; Emory University, Atlanta, Georgia, United States; Emory University School of Medicine, Atlanta, Georgia, United States; Emory, Atlanta, Georgia, United States; Emory University, Atlanta, Georgia, United States

## Abstract

Design Thinking provides an empathy-based creative problem-solving approach to intervention and/or patient-centered care design that seeks to articulate the right problem space and propose appropriate solutions to challenges encountered by individuals in specific contexts, such as healthcare. This abstract illustrates the application of the Design Thinking approach in the context of dementia caregiving. Emory’s Roybal Center for Caregiving Mastery conducted a three-part design studio workshop with African American dementia family caregivers to understand and identify prioritized solutions to their caregiving challenges. The workshops followed the Double-Diamond design process model that is divided into four phases (Discover, Define, Develop, and Deliver) and that emphasizes divergent and convergent modes of thinking. The process first encourages divergent thinking to explore a problem space and then employs convergent thinking to identify the salient problem and develop the right solution. The workshop’s Discover stage provided a broad and deep contextual understanding of the range of unmet needs African American caregivers experience in their interactions with healthcare personnel and systems. The Define phase enabled the convergent review and prioritization of the list of needs to be addressed. The Develop phase generated 17 intervention ideas. Finally, the Deliver phase focused on converging and validating the proposed interventions. The workshops concluded that having access to caregiver-focused resources supporting mental/physical self-care, such as support groups and peer programs, can be beneficial interventions to potentially improve knowledge, awareness, and caregiving mastery among African Americans American caregivers.

